# Crystal structure of bis­(pivaloyl­hydroxamato-κ^2^ *O*,*O*′)copper(II)

**DOI:** 10.1107/S2056989018012227

**Published:** 2018-08-31

**Authors:** Kateryna Goleva, Dina Naumova, Anna Pavlishchuk, Anthony W. Addison, Matthias Zeller

**Affiliations:** a Department of Chemistry, Taras Shevchenko National University of Kyiv, Volodymyrska str. 62, Kiev, 01601, Ukraine; b Department of Chemistry, Drexel University, Philadelphia, PA 19104-2816, USA; c Department of Chemistry, Purdue University, 560 Oval Dr., West Lafayette, IN 47907-2084, USA

**Keywords:** crystal structure, copper(II), hydroxamates, pivalate derivatives, mononuclear complexes

## Abstract

Reaction of copper(II) nitrate with pivaloyl­hydroxamic acid yielded the title compound, [Cu(pivHA)_2_] (where pivHA^−^ is pivaloyl hydroxamate, C_5_H_10_NO_2_). The centrosymmetric mononuclear complex consists of a Cu^II^ ion, which is located on a center of inversion, with two coordinated pivaloyl hydroxamate monoanions. The Cu^II^ ion has a square-planar coordination environment consisting of four O atoms – two carbonyl O atoms and two hydroxamate O atoms from two hydroxamate pivHA^−^ ligands. The pivHA^−^ anions are coordinated to copper(II) in a *trans*-mode, forming two five-membered *O*,*O*′-chelate rings.

## Abstract 2

The mononuclear discrete complex [Cu(pivHA)_2_] contains two pivaloyl hydroxamates (pivHA^−^) coordinated to a copper(II) ion in a centrosymmetric *trans* mode through hydroxamate and carbonyl O atoms. The copper(II) O_4_ coordination is square planar.

## Chemical context   

Numerous studies over the past decade of various hydroxamate complexes with 3*d* and 4*f* metal ions have been inspired by their potential applications in mol­ecular magnetism, luminescence, adsorption and catalysis (Ostrowska *et al.*, 2016 ▸; Pavlishchuk *et al.*, 2015 ▸). The ability of further functionalized hydroxamic acids to serve as bridging ligands and to form polynuclear species with different structural motifs has been comprehensively examined in recent years (Mezei *et al.*, 2007 ▸; Pavlishchuk *et al.*, 2018 ▸; Odarich *et al.*, 2016 ▸; McDonald *et al.*, 2014 ▸, 2015 ▸; Gaynor *et al.*, 2002 ▸). Studies of simple unsubstituted hydroxamic acids have been undertaken because of their possible application as mimics of mononuclear iron(III) siderophores (Marmion *et al.*, 2004 ▸). As a result of the potentially multiple coordination modes of unsubstituted hydroxamic acids, they can also lead to the formation of polynuclear assemblies (Tirfoin *et al.*, 2014 ▸). However, reactions of unsubstituted hydroxamic acids with transition metal ions lead mainly to the formation of octa­hedral 1:3 (Abu-Dari *et al.*, 1979 ▸) or square-planar 1:2 (Baughman *et al.*, 2000 ▸) complexes with the hydroxamate in an *O*,*O*′-coordination mode. The ability of pivalic acid itself to form polynuclear metallamacrocyclic complexes with various metal ions is well known (Vitórica-Yrezábal *et al.*, 2017 ▸; Garlatti *et al.*, 2018 ▸). The aim of the current work was to investigate if a *tert*-butyl-substituted hydroxamic acid (*i.e.* the hydroxamate analogue of pivalic acid) could be used as a scaffold for the preparation of polynuclear copper(II) complexes.

## Structural commentary   

Crystals of the title compound **1** were obtained by reaction of copper(II) nitrate hexa­hydrate with pivaloyl­hydroxamic acid in methanol.
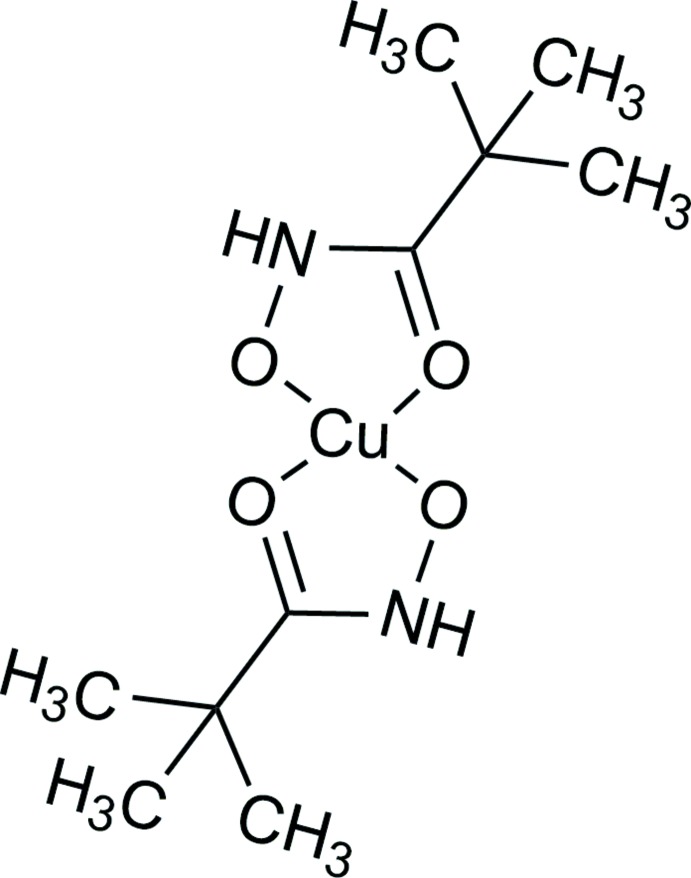


**Figure 1:**
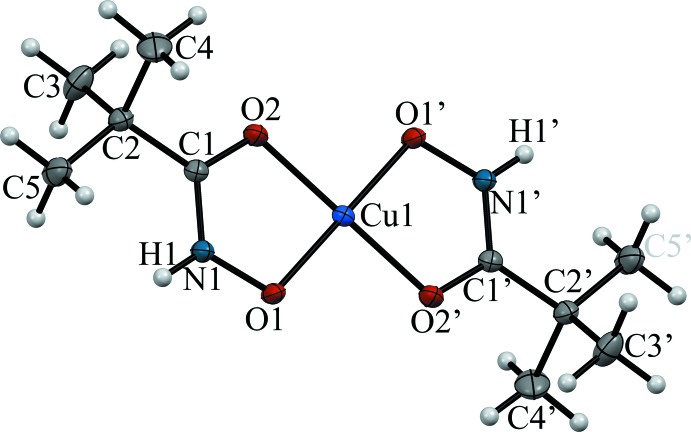
The mol­ecular structure of complex **1** showing the neutral centrosymmetric fragment [Cu(pivHA)_2_], along with the atom labelling. Displacement ellipsoids are at the 50% probability level. Symmetry code: (’) −*x*, 1 − *y*, −*z.*

Complex **1** crystallizes in the space group *I*4_1_/*a*, with eight [Cu(pivHA)_2_] complex mol­ecules per unit cell. The [Cu(pivHA)_2_] mol­ecules are centrosymmetric, with the copper ion located on an inversion center. Each [Cu(pivHA)_2_] mol­ecule contains one copper(II) ion in a square-planar coordination environment generated by the coordination of two pivaloyl­hydroxamate monoanions, forming five-membered chelate rings through both the carbonyl and hydroxamate O atoms (Fig. 1 ▸). The centrosymmetric nature of the complex forces the copper(II) ions to be exactly coplanar with the four donor O atoms, O1O2O1^i^O2^i^ [symmetry code: (i) −*x*, 1 − *y*, −*z*], and the two pivHA^−^ monoanions in [Cu(pivHA)_2_] are necessarily mutually *trans-*coordinated. The axial positions of the copper(II) ions remain unoccupied. The Cu—O_carbon­yl_ and Cu—O_hydroxamate_ bond lengths are typical for copper(II) hydroxamate or oximate complexes (Buvailo *et al.*, 2012 ▸; Pavlishchuk *et al.*, 2017*a* ▸,*b* ▸) (Table 1 ▸). The hydroxamate N—H groups remain protonated and are not involved in metal coordination. Deprotonation of the N—H groups could possibly be achieved at higher pH without hydrolysis of hydroxamic acid, which might aid in the formation of polynuclear complexes.

## Supra­molecular features   

**Figure 2:**
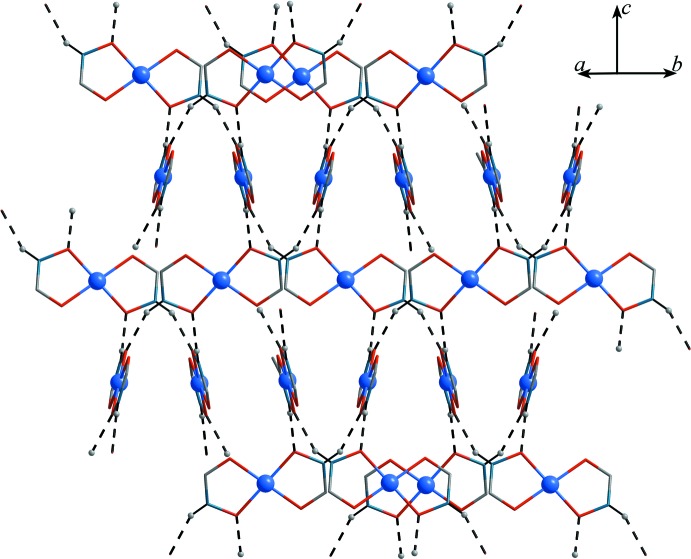
A fragment of the lattice of complex **1**, showing the intra­molecular hydrogen-bonding connections (dashed lines) between the [Cu(pivHA)_2_] mol­ecules. The *tert*-butyl groups are omitted for clarity.

**Figure 3:**
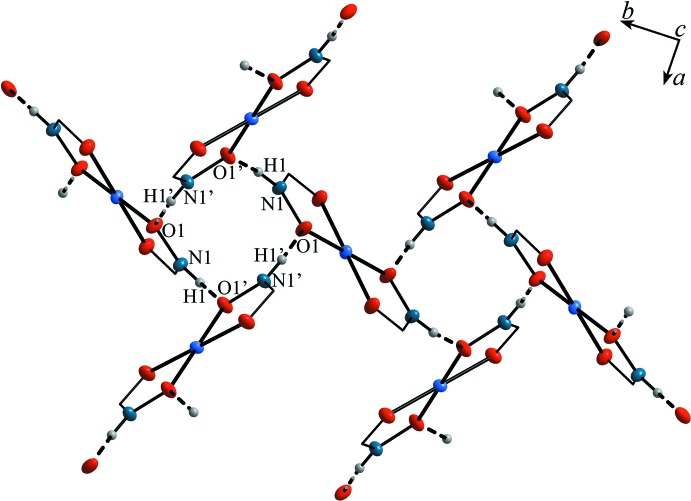
A fragment of the packing of complex **1**, showing the formation of supra­molecular tetra­mers [Cu(pivHA)_2_]_4_ formed by hydrogen bonds. The *tert*-butyl groups are omitted for clarity.

Adjacent [Cu(pivHA)_2_] complexes are connected to each other *via* N1–H1⋯O1^ii^ hydrogen bonds between the hydroxamate N—H group of one complex mol­ecule and a deprotonated hydroxamate oxygen of an adjacent [Cu(pivHA)_2_] mol­ecule (Table 2 ▸, Fig. 2 ▸). Four of these N—H⋯O hydrogen bonds connect mol­ecules into tetra­mers arranged around a fourfold rotoinversion center. The N—H group of the second hydroxamate ligand of each complex creates an equivalent tetra­mer *trans* across the copper ion, thus creating an infinite three-dimensional network of corner-connected tetra­mers (with the copper ions acting as the bridging element, Fig. 3 ▸).

## Database survey   

The Cambridge Structural Database (CSD, Version 5.27, updated in August 2012; Groom *et al.*, 2016 ▸) contains one report with structural information for pivaloyl­hydroxamic acid (CCDC 1155138; Due *et al.*, 1987 ▸). Though the survey did not contain any information about complexes with pivaloyl­hydroxamic acid, there are two reports devoted to structural studies of Th^4+^ (1180613 and 1180614; Smith & Raymond, 1981 ▸) and MoO_2_^2+^ (763210–763214; Dzyuba *et al.*, 2010 ▸) complexes with structurally similar ligands (*N*-isopropyl-2,2-di­methyl­propane­hydroxamate, *N*-isopropyl-3,3-di­methyl­butane­hydroxamate and decano-, *N*-methyl-decano-, *N*-methyl-hexano-, *N*-methyl-1-adamantano- or *N*-*tert*-butyl-hexa­nohydroxamates, respectively). It should be mentioned that coordination of hydroxamate ligands in the *O*,*O*′-chelating mode is quite typical (Tedeschi *et al.*, 2003 ▸; Seitz *et al.*, 2007*a* ▸,*b* ▸; Brewer & Sinn, 1981 ▸) and the CSD contains many records with such binding in various mononuclear bis-hydroxamate complexes (*e.g*. Drovetskaia *et al.*, 1996 ▸; Li *et al.*, 2004 ▸; Fisher *et al.*, 1989 ▸; Harrison *et al.*, 1976 ▸), which are usually coordinated in the *trans*- mode with respect to each other (Gaynor *et al.*, 2001 ▸; Lasri *et al.*, 2012 ▸; Casellato *et al.*, 1984 ▸).

## Synthesis and crystallization   

A solution of pivaloyl­hydroxamic acid (23.4 mg, 0.20 mmol) in 5 mL of methanol was added to copper(II) nitrate hexa­hydrate (29.6 mg, 0.10 mmol) in 5 mL of methanol. The resulting blue solution was stirred for 30 min. at room temperature, filtered and left for slow evaporation. After a week, blue crystals suitable for single crystal X-ray analysis had formed. Yield: 23 mg (78%). Elemental analysis C:H:N Expected (calculated): 40.75 (40.60): 7.03 (6.81): 9.22 (9.47). IR in KBr pellets (cm^−1^): 3400 (ν_N–H_); 3196–3040 (ν_O–H_, likely due to the presence of N1—H1⋯O1^ii^ hydrogen bonds); 1595 and 1503 (ν_amid I_); 1330, 1220 and 1053 (ν_C–C_ and ν_-C-N_); 963 (ν_N–O_).

## Refinement   

Crystal data, data collection and structure refinement details are summarized in Table 3 ▸. H atoms attached to carbon and nitro­gen atoms were positioned geometrically and constrained to ride on their parent atoms: C—H =0.98 Å with *U*_iso_(H) = 1.5*U*_eq_(C) and N—H = 0.88 Å with *U*_iso_(H) = 1.2*U*_eq_(N). Methyl H atoms were allowed to rotate but not to tip to best fit the experimental electron density.

## Supplementary Material

Crystal structure: contains datablock(s) I. DOI: 10.1107/S2056989018012227/ex2012sup1.cif

Structure factors: contains datablock(s) I. DOI: 10.1107/S2056989018012227/ex2012Isup2.hkl

Supporting information file. DOI: 10.1107/S2056989018012227/ex2012Isup3.cdx

Additional supporting information:  crystallographic information; 3D view; checkCIF report

## Figures and Tables

**Table 1:** Selected geometric parameters (Å, °)

C1—O2	1.2821 (13)	O1—Cu1	1.8899 (8)
C1—N1	1.3066 (14)	O2—Cu1	1.9244 (8)
N1—O1	1.3764 (12)		
			
O1—Cu1—O1^i^	180 (5)	O1—Cu1—O2^i^	95.16 (3)
O1—Cu1—O2	84.84 (3)	O1^i^—Cu1—O2^i^	84.84 (3)
O1^i^—Cu1—O2	95.16 (3)	O2—Cu1—O2^i^	180

Symmetry code: (i) 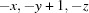.

**Table 2:** Hydrogen-bond geometry (Å, °)

*D*—H⋯*A*	*D*—H	H⋯*A*	*D*⋯*A*	*D*—H⋯*A*
N1—H1⋯O1^ii^	0.88	1.90	2.7185 (13)	154

Symmetry code: (ii) 
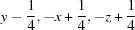
.

**Table 3:** Experimental details

Crystal data	
Chemical formula	[Cu(C_5_H_10_NO_2_)_2_]
*M*_r_	295.82
Crystal system, space group	Tetragonal, *I*4_1_/*a*
Temperature (K)	100
*a*, *c* (Å)	12.8059 (5), 17.7051 (8)
*V* (Å^3^)	2903.5 (3)
*Z*	8
Radiation type	Mo *K*α
μ (mm^−1^)	1.51
Crystal size (mm)	0.35 × 0.35 × 0.29

Data collection	
Diffractometer	Bruker D8 Quest CMOS
Absorption correction	Multi-scan (*SADABS*; Krause et al., 2015 ▸)
*T*_min_, *T*_max_	0.656, 0.747
No. of measured, independent and observed [*I* > 2σ(*I*)] reflections	24433, 2764, 2444
*R*_int_	0.035
(sin θ/λ)_max_ (Å^−1^)	0.769

Refinement	
*R*[*F* ^2^ > 2σ(*F* ^2^)], *wR*(*F* ^2^), *S*	0.029, 0.074, 1.19
No. of reflections	2764
No. of parameters	82
H-atom treatment	H-atom parameters constrained
Δρ_max_, Δρ_min_ (e Å^−3^)	0.46, −0.48

Computer programs: *APEX2* and, *SAINT* (Bruker, 2014 ▸), *SHELXS97* (Sheldrick, 2008 ▸), *SHELXL2018* (Sheldrick, 2015 ▸), *shelXle* (Hübschle *et al.*, 2011 ▸), *Mercury* (Macrae *et al.*, 2006 ▸) and *publCIF* (Westrip, 2010 ▸).

## supplementary crystallographic information

### Crystal data

**Table:**

[Cu(C_5_H_10_NO_2_)_2_]	*D*_x_ = 1.353 Mg m^−^^3^
*M**_r_* = 295.82	Mo *K*α radiation, λ = 0.71073 Å
Tetragonal, *I*4_1_/*a*	Cell parameters from 9939 reflections
*a* = 12.8059 (5) Å	θ = 3.2–33.2°
*c* = 17.7051 (8) Å	µ = 1.51 mm^−^^1^
*V* = 2903.5 (3) Å^3^	*T* = 100 K
*Z* = 8	Prism, blue
*F*(000) = 1240	0.35 × 0.35 × 0.29 mm

### Data collection

**Table:**

Bruker AXS D8 Quest CMOS diffractometer	2764 independent reflections
Radiation source: I-mu-S microsource X-ray tube	2444 reflections with *I* > 2σ(*I*)
Laterally graded multilayer (Goebel) mirror monochromator	*R*_int_ = 0.035
ω and phi scans	θ_max_ = 33.2°, θ_min_ = 3.2°
Absorption correction: multi-scan (SADABS; Krause et al., 2015)	*h* = −19→19
*T*_min_ = 0.656, *T*_max_ = 0.747	*k* = −19→19
24433 measured reflections	*l* = −27→27

### Refinement

**Table:**

Refinement on *F*^2^	Primary atom site location: structure-invariant direct methods
Least-squares matrix: full	Secondary atom site location: difference Fourier map
*R*[*F*^2^ > 2σ(*F*^2^)] = 0.029	Hydrogen site location: inferred from neighbouring sites
*wR*(*F*^2^) = 0.074	H-atom parameters constrained
*S* = 1.19	*w* = 1/[σ^2^(*F*_o_^2^) + (0.0257*P*)^2^ + 3.2993*P*] where *P* = (*F*_o_^2^ + 2*F*_c_^2^)/3
2764 reflections	(Δ/σ)_max_ < 0.001
82 parameters	Δρ_max_ = 0.46 e Å^−^^3^
0 restraints	Δρ_min_ = −0.48 e Å^−^^3^

### Special details

**Table:**

Geometry. All esds (except the esd in the dihedral angle between two l.s. planes) are estimated using the full covariance matrix. The cell esds are taken into account individually in the estimation of esds in distances, angles and torsion angles; correlations between esds in cell parameters are only used when they are defined by crystal symmetry. An approximate (isotropic) treatment of cell esds is used for estimating esds involving l.s. planes.

### Fractional atomic coordinates and isotropic or equivalent isotropic displacement parameters (Å^2^)

**Table:**

	*x*	*y*	*z*	*U*_iso_*/*U*_eq_	
C1	0.13160 (8)	0.34258 (8)	−0.02113 (6)	0.01465 (18)	
C2	0.20901 (9)	0.26772 (9)	−0.05643 (7)	0.01747 (19)	
C3	0.30109 (12)	0.33273 (12)	−0.08492 (10)	0.0327 (3)	
H3A	0.349813	0.287558	−0.112390	0.049*	
H3B	0.337056	0.364691	−0.041874	0.049*	
H3C	0.275434	0.387597	−0.118744	0.049*	
C4	0.15406 (12)	0.21475 (12)	−0.12341 (8)	0.0284 (3)	
H4A	0.130218	0.268097	−0.159159	0.043*	
H4B	0.093923	0.174854	−0.105007	0.043*	
H4C	0.203005	0.167530	−0.148793	0.043*	
C5	0.24745 (12)	0.18527 (12)	−0.00063 (8)	0.0274 (3)	
H5A	0.187982	0.144200	0.017576	0.041*	
H5B	0.281569	0.219691	0.042206	0.041*	
H5C	0.297448	0.139024	−0.025866	0.041*	
N1	0.10376 (7)	0.33335 (8)	0.04955 (5)	0.01550 (17)	
H1	0.128862	0.282995	0.078083	0.019*	
O1	0.03387 (7)	0.40497 (7)	0.07791 (5)	0.01837 (16)	
O2	0.09269 (7)	0.41676 (7)	−0.06079 (5)	0.01830 (16)	
Cu1	0.000000	0.500000	0.000000	0.01360 (6)	

### Atomic displacement parameters (Å^2^)

**Table:**

	*U*^11^	*U*^22^	*U*^33^	*U*^12^	*U*^13^	*U*^23^
C1	0.0150 (4)	0.0146 (4)	0.0143 (4)	−0.0005 (3)	0.0017 (3)	0.0009 (3)
C2	0.0180 (5)	0.0179 (5)	0.0166 (5)	0.0020 (4)	0.0038 (4)	0.0011 (4)
C3	0.0242 (6)	0.0314 (7)	0.0424 (8)	−0.0013 (5)	0.0165 (6)	0.0028 (6)
C4	0.0311 (7)	0.0303 (6)	0.0238 (6)	0.0075 (5)	−0.0015 (5)	−0.0098 (5)
C5	0.0321 (7)	0.0275 (6)	0.0227 (6)	0.0144 (5)	0.0056 (5)	0.0046 (5)
N1	0.0168 (4)	0.0156 (4)	0.0142 (4)	0.0034 (3)	0.0031 (3)	0.0026 (3)
O1	0.0227 (4)	0.0186 (4)	0.0138 (3)	0.0077 (3)	0.0063 (3)	0.0033 (3)
O2	0.0241 (4)	0.0172 (4)	0.0136 (3)	0.0045 (3)	0.0037 (3)	0.0035 (3)
Cu1	0.01670 (10)	0.01244 (9)	0.01165 (9)	0.00085 (6)	0.00182 (6)	0.00171 (6)

### Geometric parameters (Å, º)

**Table:**

C1—O2	1.2821 (13)	C4—H4B	0.9800
C1—N1	1.3066 (14)	C4—H4C	0.9800
C1—C2	1.5141 (16)	C5—H5A	0.9800
C2—C5	1.5275 (18)	C5—H5B	0.9800
C2—C3	1.5290 (18)	C5—H5C	0.9800
C2—C4	1.5368 (18)	N1—O1	1.3764 (12)
C3—H3A	0.9800	N1—H1	0.8800
C3—H3B	0.9800	O1—Cu1	1.8899 (8)
C3—H3C	0.9800	O2—Cu1	1.9244 (8)
C4—H4A	0.9800		
			
O2—C1—N1	119.04 (10)	H4A—C4—H4C	109.5
O2—C1—C2	119.84 (10)	H4B—C4—H4C	109.5
N1—C1—C2	121.12 (10)	C2—C5—H5A	109.5
C1—C2—C5	112.43 (10)	C2—C5—H5B	109.5
C1—C2—C3	107.24 (10)	H5A—C5—H5B	109.5
C5—C2—C3	109.95 (12)	C2—C5—H5C	109.5
C1—C2—C4	107.35 (10)	H5A—C5—H5C	109.5
C5—C2—C4	109.97 (11)	H5B—C5—H5C	109.5
C3—C2—C4	109.81 (11)	C1—N1—O1	117.82 (9)
C2—C3—H3A	109.5	C1—N1—H1	121.1
C2—C3—H3B	109.5	O1—N1—H1	121.1
H3A—C3—H3B	109.5	N1—O1—Cu1	108.18 (6)
C2—C3—H3C	109.5	C1—O2—Cu1	110.11 (7)
H3A—C3—H3C	109.5	O1—Cu1—O1^i^	180.00 (5)
H3B—C3—H3C	109.5	O1—Cu1—O2	84.84 (3)
C2—C4—H4A	109.5	O1^i^—Cu1—O2	95.16 (3)
C2—C4—H4B	109.5	O1—Cu1—O2^i^	95.16 (3)
H4A—C4—H4B	109.5	O1^i^—Cu1—O2^i^	84.84 (3)
C2—C4—H4C	109.5	O2—Cu1—O2^i^	180.0
			
O2—C1—C2—C5	179.56 (11)	C2—C1—N1—O1	179.33 (10)
N1—C1—C2—C5	−0.14 (16)	C1—N1—O1—Cu1	−0.49 (12)
O2—C1—C2—C3	58.59 (15)	N1—C1—O2—Cu1	1.02 (13)
N1—C1—C2—C3	−121.11 (13)	C2—C1—O2—Cu1	−178.69 (8)
O2—C1—C2—C4	−59.37 (14)	N1—O1—Cu1—O2	0.79 (7)
N1—C1—C2—C4	120.94 (12)	N1—O1—Cu1—O2^i^	−179.21 (7)
O2—C1—N1—O1	−0.37 (16)		

Symmetry code: (i) −*x*, −*y*+1, −*z*.

### Hydrogen-bond geometry (Å, º)

**Table:**

*D*—H···*A*	*D*—H	H···*A*	*D*···*A*	*D*—H···*A*
N1—H1···O1^ii^	0.88	1.90	2.7185 (13)	154

Symmetry code: (ii) *y*−1/4, −*x*+1/4, −*z*+1/4.
